# Oral health related quality of life in pregnant and post partum women in two social network domains; predominantly home-based and work-based networks

**DOI:** 10.1186/1477-7525-10-5

**Published:** 2012-01-13

**Authors:** Gabriela A Lamarca, Maria do C Leal, Anna TT Leao, Aubrey Sheiham, Mario V Vettore

**Affiliations:** 1 Escola Nacional de Saúde Pública, Fundação Oswaldo Cruz/FIOCRUZ, Rio de Janeiro, BR; 2 Department of Epidemiology and Public Health, University College London, London, UK; 3 Faculdade de Odontologia, Universidade Federal do Rio de Janeiro, Rio de Janeiro, BR; 4 Instituto de Estudos em Saúde Coletiva, Universidade Federal do Rio de Janeiro, Rio de Janeiro, BR

**Keywords:** women's health, oral health, quality of life, social support, social networks, occupation

## Abstract

**Background:**

Individuals connected to supportive social networks have better general and oral health quality of life. The objective of this study was to assess whether there were differences in oral health related quality of life (OHRQoL) between women connected to either predominantly home-based and work-based social networks.

**Methods:**

A follow-up prevalence study was conducted on 1403 pregnant and post-partum women (mean age of 25.2 ± 6.3 years) living in two cities in the State of Rio de Janeiro, Brazil. Women were participants in an established cohort followed from pregnancy (baseline) to post-partum period (follow-up). All participants were allocated to two groups; 1. work-based social network group - employed women with paid work, and, 2. home-based social network group - women with no paid work, housewives or unemployed women. Measures of social support and social network were used as well as questions on sociodemographic characteristics and OHRQoL and health related behaviors. Multinomial logistic regression was performed to obtain OR of relationships between occupational contexts, affectionate support and positive social interaction on the one hand, and oral health quality of life, using the Oral Health Impacts Profile (OHIP) measure, adjusted for age, ethnicity, family income, schooling, marital status and social class.

**Results:**

There was a modifying effect of positive social interaction on the odds of occupational context on OHRQoL. The odds of having a poorer OHIP score, ≥4, was significantly higher for women with home-based social networks and moderate levels of positive social interactions [OR 1.64 (95% CI: 1.08-2.48)], and for women with home-based social networks and low levels of positive social interactions [OR 2.15 (95% CI: 1.40-3.30)] compared with women with work-based social networks and high levels of positive social interactions. Black ethnicity was associated with OHIP scores ≥4 [OR 1.73 (95% CI: 1.23-2.42)].

**Conclusions:**

Pregnant and post-partum Brazilian women in paid employment outside the home and having social supports had better OHRQoL than those with home-based social networks.

## Introduction

Social networks and social cohesion affect health [1,2]. The perceptions of general health and overall quality of life are influenced by the received social support [3]. Individuals connected to supportive social networks have better general and oral health related quality of life (OHRQoL) [4]. The current concepts of social networks focus on how structural arrangements of social institutions shape resources available to individuals, and hence, their behavioral and emotional responses [1]. The structure of network ties influences people's health by providing different types and levels of support. Lower social support is associated with more symptoms of depression [5-8] and poor social support is linked to higher mortality rates [9-11].

Berkman and Kawachi argued that social networks operate at the behavioral level through social support and social influence, which affects social engagement and attachment and access to resources and material goods [1]. The concepts of social networks and social supports are intrinsically interconnected and overlap [12]. However, social networks are the structure through which social support is provided [13]. Social support is generally defined in terms of the availability of people who individuals trust, and on whom they can rely on and who will care for them [1]. Research on social support emphasizes the importance of types, frequency, intensity and extent of social networks and on the effects of variation of the individual's social environment [14] as well as on the contexts for developing social networks [1].

The main mechanism that might explain why social support operates via social networks and enhances quality of life is the existence of positive social relationships. Social networks can enhance mood, provide people with a sense of identity, enhance coping strategies and be a source of companionship for sharing activities [4].

Lack of social support is an important risk factor for maternal well-being and quality of life during pregnancy, and has adverse effects on pregnancy outcomes [15]. Some studies on the relationship between social support and health in pregnant women have focused on social support interventions; others were related to family support [16,17]. Women with low social support are more likely to report postnatal depression and lower quality of life than well-supported women [18]. Pregnant women with poor social networks were at high risk for emotional and behavioral problems both to mothers and their children [19].

As stated earlier, the contexts for developing social networks affect the quality and quantity of social support. Employed women are healthier than those not employed [20,21]. That suggests that work colleagues can be an important network of social relationships and social support. They are likely to confer health benefits [22]. Social processes in women's daily activities may affect their subjective perceptions of health. In a study of Japanese women workers, poor social networks at work were associated with worse self-perceived health, mainly among older women. Older workers with social networks mainly at work reported better health than those with better social networks at home [23]. Furthermore, there was a positive association between lack of social networks outside the work environment and worse general health among middle-aged women [23]. There is a positive relationship between work-related psychosocial factors such as decision latitude, job demands and social support, and the health of workers [24]. Women in the labor market may perform tasks involving high demand and over which they have little control. That may lead to stress and poorer health. In addition, they may be less intellectually and socially stimulated; aspects considered harmful to health [25,26].

Oral health conditions are associated with social networks and social support [27-29]. The use of dental services was associated with better levels of social networks and social support [27,28]. Men who had more social supports and those reporting having at least one close friend and those who participated in religious activities were less likely to develop periodontitis [30]. Whereas there are numerous studies showing that dental status affects OHRQoL [31-34], there are very few on the relationship between social networks, using social support as a measure of support, and domains of OHRQoL [35].

There are very few studies on OHRQoL in pregnant women. In two studies the prevalence of negative impacts of pregnancy on OHRQoL was about 25% [36,37]. Oral pain during pregnancy had a negative effect on women's quality of life. The most frequently mentioned effects were difficulty in maintaining emotional balance, difficulty eating and difficulty cleaning teeth [36]. As studies showed that social support during pregnancy affected their health and other outcomes, it was considered important to test whether social support from the supportive relationships in the predominant environments of pregnant women, namely home or work contexts, affected their OHRQoL. The study focused on the different domains of social support that women get predominantly from work-related networks compared to those from home-based networks, rather than on the elaboration of the structural aspects of social networks.

The objective of the main study [38], of which this is a part, was that social support and social network affect positively women's health. The specific hypothesis for this study was that predominantly home-based social network women with low social support had poorer perceived OHRQoL than those whose social networks were work-based and had high social support. The objective was to assess whether there were differences in OHRQoL between women connected to either predominantly home-based and work-based social networks. The research sets out to provide insights into the possible associations of predominantly occupational contexts, home or work, linked to social support and OHRQoL in pregnant and post-partum women.

## Methods

A follow-up prevalence study was carried out in two middle-sized cities in the State of Rio de Janeiro, Brazil, to test the relationship of social determinants with pregnancy outcomes and oral health measures [39]. All pregnant women enrolled in a fixed cohort who sought prenatal care at the four main public health care units administered by the National Health Care System ("*Sistema Unico de Saude - SUS*") were selected and invited to participate in this study. They were a representative sample of 95% of the women who were pregnant during the study period in both cities.

The sample size was estimated as 1059 subjects based on the prevalence of 59.5% of the impact of oral health on quality of life, considering OHIP > 1 [32,40] to detect a 5% of the differences between groups, with a significance level of 5% and power of 95% [41]. A study with 20% of losses during follow-up required 1270 participants.

Primary data were collected through face-to-face individual structured interviews between October 2008 and December 2009. The information was obtained at baseline (first trimester of pregnancy) and during the 30 days postpartum period (follow-up).

The selection criteria were women in the first trimester of pregnancy and living at their current address for at least 12 months. The latter criterion was used because social networks and social support tend to be stable after some months. First, the interviewers inspected the medical notes and chose pregnant women according to the selection criteria. All eligible pregnant women were invited to participate. They were informed about the objectives of the study. One of the interviewers requested their participation. After obtaining their consent, the women were interviewed. The study was approved by the Committee of Ethics and Research of the National School of Public Health - ENSP/FIOCRUZ (protocol no. 158/06).

### Definition of occupational context

The main exposure was the occupational context, which was considered to be composed of different characteristics of way of life and characteristics related to occupational status.

### Groups of comparison

Participants were allocated to two groups: 1. the work-based social network group were employed women with paid work. 2. the home-based social network group were women with no paid work, housewives or unemployed women. Measures of social support and social network were evaluated to characterize the occupational context.

### Social network and social support measures

Social networks was considered as the "web" of social relationships surrounding the individual as well as their characteristics, or groups of people who have contact with, or with some form of participation [42]. The questionnaire used to assess social networks consisted of 5 questions concerning the person's relationship with family and friends, and their participation in social groups. The instrument has adequate psychometric properties for the Brazilian population [43,44]. Social support was considered as a system of formal and informal relationships through which individuals receive emotional support, material or information to cope with stressful emotional situations [45]. Social support was evaluated using a questionnaire consisting of 19 items comprising five dimensions of functional social support: material (4 questions - provision of practical resources and support material), emotional (3 questions - physical expressions of love and affection), emotional (4 questions - expressions of positive affection, understanding and feelings of confidence), positive social interaction (4 questions - availability of people to have fun or relax), and information (4 questions - availability of people to obtain advice or guidance) [14]. For each item, the women indicated how often they experienced each type of available support: never, rarely, sometimes, often or always. This questionnaire had good reliability for the Brazilian population [44].

### The impact of oral health on quality of life

The outcome was the impact of oral health on quality of life, which reflects the perception of people about dysfunction, discomfort and disability related oral conditions. The validated version of Oral Health Impacts Profile (OHIP-14) for Brazilian population was used to evaluate the experience of impact on oral health on quality of life in the preceding 6 months [32,40]. OHIP-14 is composed of 14 items, aggregated in 7 dimensions (two items per dimension) as following: functional limitation (items 1 and 2), physical pain (items 3 and 4), psychological discomfort (items 5 and 6), physical disability (items 7 and 8), psychological disability (items 9 and 10), social disability (items 11 and 12) and handicap (items 13 and 14). The overall score was computed by additive method, which is the sum of the individual scores of all items. For each item, the score varied from 0 to 4: "never" = 0, "hardly ever" = 1, "occasionally" = 2, "often" = 3, and "very often" = 4. A high score indicates a negative influence of oral health on quality of life.

### Covariates

The covariates were demographic and socioeconomic characteristics, health related behaviors previous and during pregnancy, dental pain in the last 6 months and number of teeth (<10 teeth versus ≥10 teeth). Demographic data were maternal age, ethnicity and number of children.

Socioeconomic characteristics were marital status, educational level (years of schooling), familial income, head of the family, housing conditions and social class. In this study the term social class refers to the social and economic factors that influence what position(s) individuals and groups hold within the structure of society [46]. A standard social class classification commonly used in Brazil was used [47]. This is an economic classification based on market power comprising a group of specific indicators such as number of bathrooms, number of full-time domestic servants, number of cars owned by the family, possession of domestic items such as television sets, radio sets, VCRs, vacuum cleaners, washing machine, fridges, freezers; and level of education of the head of household. A set of points is assigned to these indicators and a final score defines the socioeconomic groups; A (highest), B, C, D, and E (lowest). Those with the highest scores represented the highest socioeconomic groups.

The health behaviors, assessed before pregnancy, were smoking, cigarette consumption and alcohol consumption. In addition, the Brazilian version of T-ACE questionnaire, based on 5 questions concerning self-perception of drinking habits, was used to assess risky alcohol drinking before pregnancy [48].

### Pilot study

The interviewers were trained to conduct structured and standardized interviews. After training the interviewers, a pilot study was performed to test understanding and layout of questionnaires. Examiners interviewed 40 pregnant selected women at the same health care units of the main study but who were not included in the main study.

### Main study

Data collection was performed by 20 trained interviewers and four fieldwork supervisors. The baseline was conducted in the prenatal health care units to collect occupational context data, social network, social support, demographic and socioeconomic characteristics, number of teeth and health related behaviors. During the baseline interview different strategies were established to reduce the losses to follow-up. First, two telephone numbers were requested. Second, the full current address was registered, including the zip code. Third, contact telephone numbers of the fieldwork supervisors were provided for all women. They were requested to telephone one of the supervisors when admission to the maternity unit or discharge from it was arranged. In addition, they were asked to report if they moved home or changed their telephone number.

The follow-up study was performed in the post partum period immediately after the delivery to collect data on the impact of oral health on quality of life and dental pain in the last 6 months. The interview was conducted in the maternity hospital wards or at the mother's house up to 30 days after discharge. Women who moved home were excluded. In addition, those who had a miscarriage (pregnancy interrupted before the 20^th ^gestational week) or abortion were not re-interviewed.

### Data analysis

All variables were computed for each participant and then for each group. The normal distribution of continuous variables was tested using the Kolmogorov-Smirnov test. Since the continuous variables were not normally distributed, the comparison of groups was performed by Mann-Whitney test. Categorical variables were analyzed by Chi-square test.

Internal consistencies for the OHIP scale and its domains were evaluated by the Cronbach's α coefficient. Cronbachs' α removing each domain of the OHIP were also assessed.

The relationship between occupational context and the impact of oral health on quality of life was tested using multinomial logistic regression. The sample was categorized into 3 groups according to the prevalence and the median (the median of OHIP = 3) of the number of impacts of OHIP: OHIP = 0 (No impact); OHIP 1-3 (Scores from 1 to 3); OHIP ≥4 (Scores ≥4). In addition, the sample was grouped concerning the dimensions of social support. Subjects with low levels of impacts were those with scores equal to zero, moderate level subjects were those between zero and the median, and high level subjects were those above the median.

First, a comparison was made between social support dimensions and types of social networks in the work-based and home-based groups. Social support and social network variables that were statistically different between occupational context groups were included in the bivariate analysis. The crude Odds Ratio (OR) and Confidence Intervals of 95% were calculated between occupational context and covariates and OHIP groups. Second, multinomial logistic regression was performed to obtain adjusted OR of occupation context, affectionate support, positive social interaction and social network/friends with OHIP adjusted for age, ethnicity, family income, schooling, marital status and social class (Model 1).

To test the statistical significance of interaction between occupational context and potential modifying factors (social support dimensions and social network) the occupational context and covariates were first added to the regression model. After that, the interaction terms 'occupational context X affectionate support','occupational context X positive social interaction' and 'occupational context X social network/friends' where added to the model (Model 2). Model 1 (without interaction terms) and Model 2 (with interaction terms) were compared using Likelihood Ratio tests.

All statistical analyses were performed using the SPSS (Statistical Package for Social Sciences, version 13.0). The significance level for all analysis was 5% (P = 0.05).

## Results

Initially 1750 pregnant women were invited to participate. The acceptance rate was 96%. Of the 1680 women interviewed at baseline, 12 (0.7%) declined to participate in the follow-up, 160 (9.5%) were excluded because they had moved home and 105 (6.3%) were lost in the follow-up (miscarriage or moved home without informing the fieldwork supervisor). The final sample was 1403 women, 83.5% of the baseline sample.

Of the 1403 women, 580 (41.3%) were women in paid employment (work-based social network group) and 823 (58.7%) were unemployed women or those not doing paid work (home-based social network group). Among the women in paid work, 25 (4.3%) were civil servants, 342 (59.0%) were employees, 210 (36.2%) were self-employed, and only 3 (0.5%) were employers. Demographic data, socioeconomic and housing conditions characteristics of the occupational context groups are presented in Table 1. The average age of the sample was 25.2 ± 6.3 years; 42.8% were Brown. The participants were predominantly from low socioeconomic status, married (70.6%) and in their first pregnancy (47.3%). Even though most were living in adequate housing conditions, 42.8% reported lack of sewage and 18.4% had water supply outside the house.

**Table 1:** Demographic and socioeconomic characteristics; comparisons between work-based and home-based groups

	Work-based	Home-based	Total	P value
	N = 580	N = 823	N = 1403	
Age, M (*SD*) ^a^	26.76 ± 6.07	24.07 ± 6.22	25.18 ± 6.299	< 0.001
				
Ethnicity ^b^				0.120
White, *n *(*%*)	199 (34.5)	275 (33.5)	474 (33.9)	
Brown, *n *(*%*)	230 (39.9)	369 (44.9)	599 (42.8)	
Black, *n *(*%*)	147 (25.5)	178 (21.7)	325 (23.2)	
				
Years of schooling, M (*SD*) ^a^	8.28 ± 2.90	7.42 ± 2.91	7.78 ± 2.94	< 0.001
				
Family income ^b^				< 0.001
< 1 Minimal wage, *n *(*%*)	103 (17.8)	302 (36.7)	405 (28.9)	
1-2 Minimal wages, *n *(*%*)	199 (34.3)	250 (30.4)	449 (32.0)	
> 2 Minimal wages, *n *(*%*)	278 (47.9)	271 (32.9)	549 (39.1)	
				
Marital status ^b^				0.003
Married, living with partner, *n *(*%*)	434 (74.8)	557 (67.7)	991 (70.6)	
Has a partner, not living with him, *n *(*%*)	111 (19.1)	222 (27.0)	333 (23.7)	
Single without partner, *n *(*%*)	35 (6.0)	44 (5.3)	79 (5.6)	
				
Social Class ^b^				< 0.001
B, *n *(*%*)	40 (6.9)	43 (5.2)	83 (5.9)	
C, *n *(*%*)	399 (68.8)	480 (58.3)	879 (62.7)	
D, *n *(*%*)	122 (21.0)	251 (30.5)	373 (26.6)	
E, *n *(*%*)	19 (3.3)	49 (6.0)	68 (4.8)	
				
Head of family ^b^				< 0.001
Woman, *n *(*%*)	113 (19.5)	48 (5.8)	161 (11.5)	
Husband or partner, *n *(*%*)	341 (58.9)	485 (58.9)	826 (58.9)	
Other, *n *(*%*)	120 (20.7)	282 (34.3)	402 (28.7)	
				
Number of children ^b^				< 0.001
No children, *n *(*%*)	249 (42.9)	415 (50.4)	664 (47.3)	
1 child, *n *(*%*)	203 (35.0)	208 (25.3)	411 (29.3)	
2 or more children, *n *(*%*)	128 (22.1)	200 (24.3)	328 (23.4)	
				
Sewage in your house ^b^				0.016
Lack of sewage or pit sewage, *n *(*%*)	226 (39.0)	374 (45.4)	600 (42.8)	
General drainage, *n *(*%*)	354 (61.0)	449 (54.6)	803 (57.2)	
				
Number of residents per room ^b^				< 0.001
1, *n *(*%*)	233 (40.2)	240 (29.2)	473 (33.7)	
2, *n *(*%*)	244 (42.1)	396 (48.1)	640 (45.6)	
3, *n *(*%*)	72 (12.4)	124 (15.1)	196 (14.0)	
> 3, *n *(*%*)	31 (5.3)	63 (7.7)	94 (6.7)	
				
Water supply to house ^b^				0.352
Water plumbing supply inside the house, *n *(*%*)	480 (82.8)	665 (80.8)	1145 (81.6)	
Water plumbing supply outside the house, *n *(*%*)	100 (17.2)	158 (19.2)	258 (18.4)	

^a ^Mann-Whitney test ^b ^Chi-square test

Women from the work-based social network group were older, had more years of schooling and higher family income compared with women in the home-based social network group. The work-based social network group had more married women, and women who were head of family and from higher (B and C) social classes. The home-based social network group had a higher proportion of women living in houses without general drainage (P < 0.001) and more residents per room (P < 0.001) (Table 1).

The comparison between oral health measures and health related behaviors in work-based women and those with home-based social networks is presented in Table 2. Women from the work-based social network group had lower OHIP-14 scores than those from home-based social network group (3.5 versus 4.0), but the statistical significance was borderline. There was no difference in the proportions of women in the two groups with dental pain in the last six months and with 10 teeth or more. The frequency of alcohol intake, alcoholism and smoking was similar in the two groups (Table 2).

**Table 2:** Oral health measures and health related behaviors; comparisons between work-based and home-based groups

	Work-based	Home-based	Total	P value
	N = 580	N = 823	N = 1403	
**Oral Health Measures**				
				
OHIP, M (*SD*) ^a^	3.47 ± 7.29	3.97 ± 7.60	3.76 ± 7.47	0.059
				
Dental pain in last 6 months ^b^				0.142
No, *n *(*%*)	364 (68.5)	482 (64.6)	846 (66.2)	
Yes, *n *(*%*)	167 (31.5)	264 (35.4)	431 (33.8)	
				
Number of teeth ^b^				0.259
< 10 teeth, *n *(*%*)	21 (3.6)	40 (4.9)	61 (4.4)	
≥ 10 teeth, *n *(*%*)	558 (96.4)	780 (95.1)	1338 (95.6)	
				
**Health related behaviors**				
				
Alcohol consumption ^b^				0.213
Do not drink alcohol, *n *(*%*)	544 (93.8)	751 (91.3)	1295 (92.3)	
No risk of alcoholism, *n *(*%*)	25 (4.3)	50 (6.1)	75 (5.3)	
Risk of alcoholism, *n *(*%*)	11 (1.9)	22 (2.7)	33 (2.4)	
				
Smoking ^b^				0.061
No, *n *(*%*)	484 (83.4)	654 (79.5)	1138 (81.1)	
Yes, *n *(*%*)	96 (16.6)	169 (20.5)	265 (18.9)	
				
Number of cigarettes/day, M (*SD*) ^a^	7.27 ± 8.01	10.56 ± 12.93	9.35 ± 11.44	0.136

^a ^Mann-Whitney test ^b ^Chi-square test

There were marked differences in the social support dimensions between occupational context groups (Table 3). Affectionate support and positive social interaction scores were statistically higher in the work-based social network group compared with those in the home-based social network (P < 0.005). There was a borderline association between emotional support and informational support and being in the work-based social network group. Material support scores were similar in the two groups. Different types of social network were assessed. Women in the work-based social network group were more likely to have more friends that they felt comfortable with and could talk to about something (P = 0.001). The work-based social network group tended to have a higher proportion of women who participated in religious activities in the past 12 months (P = 0.064). Other types of social networks did not differ between groups (Table 3).

**Table 3:** Comparison of social support dimensions and types of social networks between work-based and home-based groups

	Work-based	Home-based	Total	P value
	N = 580	N = 823	N = 1403	
**Social support dimensions**				
Affectionate support, M (*SD*) ^a^	93.9 ± 12.9	91.8 ± 14.9	92.7 ±14.1	0.002
Emotional support, M (*SD*) ^a^	62.2 ± 20.2	60.0 ± 21.2	61.5 ± 20.1	0.068
Information support, M (*SD*) ^a^	62.5 ± 19.9	60.8 ± 20.2	61.5 ± 20.1	0.075
Positive social interaction, M (*SD*) ^a^	66.9 ± 17.5	62.9 ± 20.0	64.6 ± 19.1	< 0.001
Material support (tangible), M (*SD*) ^a^	59.9 ± 20.4	59.2 ± 21.2	59.5 ± 20.9	0.550
**Social network **^b^				
Relatives, *n *(*%*)	442 (81.4)	625 (82.3)	1067 (82.0)	0.662
Friends, *n *(*%*)	346 (63.7)	412 (54.3)	758 (58.2)	0.001
Meetings, *n *(*%*)	32 (5.9)	39 (5.1)	71 (5.5)	0.554
Charity work, *n *(*%*)	23 (4.2)	28 (3.7)	51 (3.9)	0.616
Religious, *n *(*%*)	380 (70.0)	494 (65.1)	874 (67.1)	0.064

^a ^Mann-Whitney test ^b ^Chi-square test

The mean OHIP-14 was 3.8 ± 7.5. The Cronbach α coefficient of OHIP-14 was 0.92. Cronbach α coefficients if OHIP-14 dimensions deleted varied from 0.90 to 0.92. The OHIP-14 scores were statistically associated with dental pain in the last six months (P < 0.05), and were not associated with the presence of 10 teeth or more.

The association of home-based social network, demographic and socioeconomic characteristic with the impact of oral health on quality of life was initially tested using unadjusted risk estimates [Odds Ratio (OR)] (Table 4). In that analysis, women with OHIP = 0 (Control group) were the reference category, and the increased odds of having a OHIP score of 1-3 (Group 1) and OHIP score ≥4 (Group 2) was estimated. The frequencies of women with OHIP score 1-3 and OHIP score ≥4 were higher in the home-based social network group compared with those in the work-based social network group (60.7% versus 39.3% and 62.5% versus 37.5%). Occupational context, affectionate support, positive social interaction, social network/friends, demographic and socioeconomic characteristics were not associated with OHIP scores 1-3. Women with home-based social networks had significantly higher odds of OHIP score ≥4 [OR 1.32 (95% CI: 1.02 - 1.70)]. Factors associated with OHIP score ≥4 were low family income, Black ethnicity and low social class. The odds of OHIP score ≥4 were significantly higher for women with low levels of affectionate support [OR 1.68 (95% CI: 1.21 - 2.33)], low positive social interaction [OR 1.71 (95% CI: 1.25 - 2.35)], family income of two minimal wages or lower [OR 1.35 (95% CI: 1.04 - 1.74)]. Black ethnicity was related to an increased chance of an OHIP score ≥4 [OR 1.76 (95% CI: 1.26 - 2.45)]. Social class level D increased the odds of OHIP score ≥4 [OR 1.95 (95% CI: 1.03-3.67)] and women in social class level E were 2.52 times more likely to have OHIP scores ≥4 (95% CI: 1.14 - 5.61) (Table 4).

**Table 4:** Crude associations between occupational context, social support dimensions, demographic and socioeconomic characteristics and OHIP

	OHIP = 0 (Reference category)	OHIP = 1-3	Crude OR CI95%	P value	OHIP ≥ 4	Crude OR CI95%	P value
Occupational context ^a^							
Work-based, *n *(%)	363 (43.8)	66 (39.3)	1		114 (37.5)	1	0.036
Home-based, *n *(%)	466 (56.2)	102 (60.7)	1.13(0.77-1.66)	0.530	190 (62.5)	1.32 (1.02-1.70)	
							
Social Support							
Affectionate support ^a^							
High level, *n *(%)	114 (13.8)	23 (18.4)	1		75 (21.6)	1	
Moderate level, *n *(%)	140 (16.9)	18 (14.4)	0.88 (0.51-1.51)	0.639	47 (13.5)	0.86 (0.60-1.23)	0.405
Low level, *n *(%)	574 (69.3)	84 (67.2)	1.38 (0.34-2.28)	0.211	225 (64.9)	1.68 (1.21-2.33)	0.002
							
Positive social interaction ^a^							
High level, *n *(%)	176 (21.3)	26 (20.8)	1		101 (29.1)	1	
Moderate level, *n *(%)	279 (33.7)	51 (40.8)	1.42 (0.93-2.17)	0.104	121 (34.9)	1.29 (0.96-1.74)	0.086
Low level, *n *(%)	373 (45.0)	48 (38.4)	1.15 (0.69-1.91)	0.596	125 (36.0)	1.71 (1.25-2.35)	< 0.001
							
Social network/Friends ^a^							
No friends, *n *(%)	358 (43.2)	44 (35.2)	1	0.093	142 (40.9)	1	0.474
One or more friends, *n *(%)	471 (56.8)	81 (64.8)	0.72 (0.48-1.06)		205 (59.1)	0.91 (0.71-1.18)	
							
Age ^a^							
13 to 24, *n *(%)	439 (53.0)	92 (54.8)	1		141 (46.4)	1	
25 to 28, *n *(%)	390 (47.0)	76 (45.2)	1.07(0.74-1.56)	0.714	163 (53.6)	1.19(0.92-1.52)	0.183
							
Schooling ^a^							
= 9, *n *(%)	348 (42.0)	79 (47.0)	1		124 (40.8)	1	
0 to 8, *n *(%)	481 (58.0)	89 (53.0)	1.09(0.74-1.59)	0.670	180 (59.2)	1.03 (0.80-1.33)	0.831
							
Familiar income ^a^							
> 2 Minimal wages, *n *(%)	439 (53.0)	76 (60.8)	1		209 (60.2)	1	
= 2 Minimal wages, *n *(%)	390 (47.0)	49 (39.2)	1.38 (0.94-2.02)	0.102	138 (39.8)	1.35 (1.04-1.74)	0.022
							
Ethnicity ^a^							
White, *n *(%)	297 (36.0)	49 (29.2)	1		87 (28.7)	1	
Brown, *n *(%)	345 (41.8)	81 (48.2)	1.44 (0.94-2.20)	0.097	128 (42.2)	1.28 (0.95-1.73)	0.110
Black, *n *(%)	183 (22.2)	38 (22.6)	0.87 (0.50-1.53)	0.638	88 (29.0)	1.76 (1.26-2.45)	0.001
							
Marital status ^a^							
Married living with partner, *n *(%)	579 (69.8)	113 (67.3)	1		230 (75.7)	1	
Married not living with partner, *n *(%)	205 (24.7)	44 (26.2)	1.17 (0.76-1.80)	0.472	59 (19.4)	0.75 (0.55-1.02)	0.064
Single, *n *(%)	45 (5.4)	11 (6.5)	1.41 (0.67-3.00)	0.368	15 (4.9)	0.84 (0.47-1.49)	0.548
							
Social Class ^a^							
B, *n *(%)	53 (6.4)	10 (6.0)	1		11 (3.6)	1	
C, *n *(%)	533 (64.3)	112 (66.7)	1.19 (0.53-2.71)	0.673	175 (57.6)	1.44 (0.78-2.66)	0.240
D, *n *(%)	210 (25.3)	39 (23.2)	1.05 (0.43-2.52)	0.921	98 (32.2)	1.95 (1.03-3.67)	0.039
E, *n *(%)	33 (4.0)	7 (4.2)	1.15 (0.34-3.91)	0.826	20 (6.6)	2.52 (1.14-5.61)	0.023

a Chi-square test

The results of the final model (Model 2) of the multinomial regression analysis of the association between occupational context and independent variables with the impact of oral health on quality of life are presented in Table 5. In the fully adjusted model (Model 1), social network/friends was not associated with home-based social network. Although social network/friends was not statistically associated with home-based social network, the interaction term 'occupational context X social network/friends' was also tested and no association with home-based social network was detected. The association of home-based social network [OR 1.34 (95% CI: 0.98-1.85)] and moderate positive social interaction [OR 1.31 (95% CI: 0.99-1.72)] with OHIP score ≥4 was of borderline significance. Low positive social interaction [OR 1.51 (95% CI: 1.01-2.27)] was significantly associated with OHIP score ≥4 prior to adding the interaction terms (occupational context X positive social interaction). The interaction term when added to this model (Model 2) was significant, suggesting that positive social interaction modified the occupational context: OHIP relationship (Table 5). Women with work-based social networks and moderate levels of positive social interaction were 1.98 more likely to have OHIP score 1-3 compared with women with work-based social networks and high levels of positive social interaction (95% CI: 1.02-3.83). The odds of OHIP score ≥4 were significantly higher for women with home-based social networks and moderate levels of positive social interaction [OR 1.64 (95% CI: 1.08-2.48)], and for women with home-based social networks and low levels of positive social interactions [OR 2.15 (95% CI: 1.40-3.30)] compared with women with work-based social networks and high levels of positive social interaction. Black ethnicity remained associated with OHIP scores ≥4 [OR 1.73 (95% CI: 1.23-2.42)]. Model 2 (with interaction terms) was statistically different from Model 1 (without interaction terms) (Chi-Square 18.827, P value = 0.043).

**Table 5:** Adjusted associations^a ^between occupational context and levels of positive social interaction, and ethnicity characteristics and OHIP

	**OHIP = 1-3 **^**b**^	P value	**OHIP ≥ 4 **^**b**^	P value
Occupational context &				
Positive social interaction (PI)				
Work-based + High level PI	1		1	
Work-based + Moderate level PI	1.98 (1.02-3.83)	0.043 *	1.35 (0.85-2.14)	0.206
Work-based + Low level PI	1.66 (0.73-3.80)	0.228	1.70 (0.98-2.86)	0.062
Home-based + High level PI	1.58 (0.85-2.94)	0.148	1.34 (0.90-2.03)	0.162
Home-based + Moderate level PI	1.75 (0.93-3.29)	0.081	1.64 (1.08-2.48)	0.020 *
Home-based + Low level PI	1.33 (0.65-2.73)	0.432	2.15 (1.40-3.30)	< 0.001 *
				
Ethnicity				
White	1		1	
Brown	1.43 (0.93-2.19)	0.104	1.23 (0.91-1.70)	0.186
Black	0.86 (0.49-1.51)	0.603	1.73 (1.23-2.42)	0.001 *

^**a **^Adjusted for schooling, age, family income, ethnicity, marital status, social class and social network/friends**.

^**b **^Reference category OHIP = 0.

* P < 0.05

** P > 0.05

## Discussion

The main finding of this study was the positive association between work-based social networks and better oral health quality of life. The identified interaction between occupational context and social support also showed a gradient in the final model of OHIP. The lower the social support, the higher the odds of having more negative oral impacts on quality of life. In addition, home-based social network women with moderate positive social interaction had significantly higher odds of having poorer oral health quality of life.

The stratified analysis illustrated the modifying effect of social support. The odds of occupational context on OHIP was higher among women with higher levels of positive social interaction compared with those with lower levels of positive social interaction. It appears that in women with high social support, the importance of occupational context on oral health is more relevant than for those with low social support. This study shows that being employed is not a sufficient condition for having lower impacts on oral health on quality of life. A combination of a higher social support (positive social interaction) and work-based social network appears to be needed to have better OHRQoL.

The observed link between occupational context and social support with oral health was probably related to the marked differences in two social support dimensions in occupational context groups. Higher scores of affectionate support and positive social interaction, domains of social support, were positively associated with predominantly work-based social network women. Even though the findings reflect and reinforce the theory that work improves and facilitates formation of stable social relationships, social support acted as an effect modifier on the relationship between occupational context and OHIP [49]. Based on the theory of benefits of work on health, social support originating from partnerships at work can provide benefits to health and decrease risks of diseases [50]. It has been hypothesized that work environment can offer greater opportunities to build self-esteem and improve confidence in the decision making processes. Employed female workers also have more social support and working increases experiences that enhances satisfactions with life [50].

The positive or negative impacts of formal work on physical and mental health are still a subject of debate. In general health, the relationship between work-related psychosocial factors, such as job control, job strain (high demand and low control), insecurity, and social support and workers' health has been widely reported [51-55]. Most of studies have focused on the association between health and the ways that work is structured in terms of hours of work, continuing education and flexibility to manage work and home demands [24]. On the other hand, occupations with high strain and lack of support at work were closely associated with psychological distress [54]. Concerning oral health outcomes, Marcenes and Sheiham (1992) addressed the relationship between work-related mental demand and periodontal disease [56]. The association of flexibility in working hours with oral health related behaviors and gingival health has also been investigated [57].

Previous evidence suggests that social connections are powerful predictors, and probably affects subjective well-being [58]. The OHIP-14 questionnaire was used as a subjective measure of the impacts of oral disorders and conditions on quality of life. The OHIP aims to evaluate the positive and negative impact of oral health on well-being, considering the social, psychological and biological dimensions. OHIP has been widely used in oral epidemiology studies to evaluate subjective oral health [59]. Our findings highlight the importance of the extent to which oral health problems are experienced by women in different occupational contexts. This study suggests possible mechanisms of how social connections and social support are important and may influence women's quality of life. Our findings agree with those of Hanson *et al. *(1994), who found that oral health related conditions were associated with social support [29]. Unfavorable socioeconomic circumstances have been associated with poor oral health outcomes regardless the indicator used or the level of analysis [60].

Paid work is the key mechanism through which people obtain important material resources to health, especially income, which in turn, is related to better diet, adequate housing and other material goods [61,62]. In this study, home-based social network women had lower levels of family income and poorer social network compared with work-based social network women. It appears that predominantly home-based women did not have enough social networks to provide sufficient social support. We can hypothesize that home-based social network women are clustered in socially excluded groups; low income and less educated women. Because of that, they would have a higher OHIP. However, it can also be argued that the poor oral health quality of life might exclude them from the labor market, thereby excluding them from social interactions, and as a result, provide less social support.

The positive aspects of the present study were the use of questionnaires with adequate psychometric properties for the Brazilian population concerning social support, social network and the impact of oral conditions on quality of life. OHIP presented good psychometrics properties. Furthermore, the data collection was standardized and collected by trained interviewers. In addition, the response rate was high and losses to follow-up were low. The time sequence of the exposure and outcome in this study provides relevant evidence on the potential benefit of work related social networks on oral health.

Although a robust sample was used in this study, our findings are limited to pregnant and post-partum women. Previous studies have shown that social support is higher in pregnant and post-partum women compared to general women in general [16,17]. In addition, our findings suggest that social support (positive social interaction) mediates the association between home-based social network and OHIP scores. Therefore, the results should not be generalized.

There is scope for more comprehensive studies on the relationship between work-based social networks and oral health. Detailed information concerning work environment should be collected in future studies, including job quality, hours spending at work and job demand. As the women in the labor market usually perform tasks over which they have little control and high demand, it would be relevant to consider women's sense of coherence in future investigations [50]. Even though paid work had a positive association with oral health, future studies can offer a better understanding about social networks at work and health. For example, the levels of social networks may vary among different types of jobs, and coping strategies may play an important role on health among those under worst job conditions or in workers with low social network at labor market.

## Conclusions

Being in paid employment and having good social support was positively related to oral health related quality of life of women during pregnancy and the post-partum period.

## Competing interests

The authors declare that they have no competing interests.

## Authors' contributions

GL was involved in design of the study, acquisition of data, analysis and interpretation of the data, interpretation of the results and drafted the manuscript. M do CL helped design the study, interpreted the data and reviewed the manuscript. ATTL was involved in the analysis and interpretation of data and contributed to writing the manuscript. AS was involved with interpretation of the data and revising the manuscript. MV was involved with the conception and design of the study, developed the statistical framework for data analysis, and drafted the manuscript. All authors read and approved the final manuscript.
